# Adaptive Interactions of *Achromobacter* spp. with *Pseudomonas aeruginosa* in Cystic Fibrosis Chronic Lung Co-Infection

**DOI:** 10.3390/pathogens10080978

**Published:** 2021-08-03

**Authors:** Angela Sandri, Janus Anders Juul Haagensen, Laura Veschetti, Helle Krogh Johansen, Søren Molin, Giovanni Malerba, Caterina Signoretto, Marzia Boaretti, Maria M. Lleo

**Affiliations:** 1 Department of Diagnostics and Public Health, Section of Microbiology, University of Verona, Strada Le Grazie 8, 37134 Verona, Italy; angela.sandri@univr.it (A.S.); caterina.signoretto@univr.it (C.S.); marzia.boaretti@univr.it (M.B.); 2 Novo Nordisk Foundation Center for Biosustainability, Technical University of Denmark, 2800 Kgs. Lyngby, Denmark; jajh@biosustain.dtu.dk (J.A.J.H.); sm@bio.dtu.dk (S.M.); 3 Laboratory of Computational Genomics, Department of Neurosciences, Biomedicine and Movement Sciences, University of Verona, 37134 Verona, Italy; laura.veschetti@univr.it (L.V.); giovanni.malerba@univr.it (G.M.); 4 Department of Clinical Microbiology, Rigshospitalet, 2100 Copenhagen, Denmark; hkj@biosustain.dtu.dk; 5 Department of Clinical Medicine, Faculty of Health and Medical Sciences, University of Copenhagen, 2200 Copenhagen, Denmark

**Keywords:** inter-species interactions, *Achromobacter* spp., *Pseudomonas aeruginosa*, lung infection, cystic fibrosis

## Abstract

In the lungs of patients with cystic fibrosis (CF), the main pathogen *Pseudomonas aeruginosa* is often co-isolated with other microbes, likely engaging in inter-species interactions. In the case of chronic co-infections, this cohabitation can last for a long time and evolve over time, potentially contributing to the clinical outcome. Interactions involving the emerging pathogens *Achromobacter* spp. have only rarely been studied, reporting inhibition of *P. aeruginosa* biofilm formation. To evaluate the possible evolution of such interplay, we assessed the ability of *Achromobacter* spp. isolates to affect the biofilm formation of co-isolated *P. aeruginosa* strains during long-term chronic co-infections. We observed both competition and cohabitation. An *Achromobacter* sp. isolate secreted exoproducts interfering with the adhesion ability of a co-isolated *P. aeruginosa* strain and affected its biofilm formation. Conversely, a clonal *Achromobacter* sp. strain later isolated from the same patient, as well as two longitudinal strains from another patient, did not show similar competitive behavior against its *P. aeruginosa* co-isolates. Genetic variants supporting the higher virulence of the competitive *Achromobacter* sp. isolate were found in its genome. Our results confirm that both inter-species competition and cohabitation are represented during chronic co-infections in CF airways, and evolution of these interplays can happen even at the late stages of chronic infection.

## 1. Introduction

Development of chronic lung infections and progressive inflammation is the major cause of morbidity and ultimate mortality for patients with cystic fibrosis (CF) [1]. Colonization with *Pseudomonas aeruginosa*, the most common pathogen isolated from CF airways, is particularly difficult to eradicate and is associated with an accelerated decline in lung function, with a poor prognosis [2]. Other respiratory pathogens play a role at different stages of the lung disease: *Staphylococcus aureus* and *Haemophilus influenzae* are the main pediatric pathogens, while *Burkholderia cepacia* complex, *Achromobacter* spp., *Stenotrophomonas maltophilia* and nontuberculous mycobacteria are mainly found in adults [3]. In particular, in the last decade, *Achromobacter* spp. gained attention as important emerging pathogens that can cause severe chronic infections in CF patients, associated with lung inflammation and decline in respiratory function [4,5,6,7,8,9] and further complicated by their innate and acquired multidrug resistance hindering eradication therapies [10,11]. The *Achromobacter* genus comprises 22 species [12]; *Achromobacter xylosoxidans* is the most often isolated species among CF patients, followed by *Achromobacter ruhlandii*, *Achromobacter insuavis*, *Achromobacter insolitus*, *Achromobacter dolens*, *Achromobacter agrifaciens* and *Achromobacter spanius* [7,13,14,15,16,17].

Due to the polymicrobial nature of CF lung infection, it is likely that microbes could engage in inter-species interactions, acting competitively or synergistically with each other to gain an adaptive advantage, thereby influencing the community composition, resistance to antibiotics and the course of airway disease [18,19]. In the case of chronic co-infections, this cohabitation can last for a long time and likely evolve. Interactions are usually favored by microbial proximity promoted by intra- and inter-species co-aggregation in biofilm communities [20,21]. The biofilm mode of growth, typical of CF chronic infections, allows bacteria to form highly organized, structured aggregates attached on the epithelial surface that protect the community from mechanical forces and penetration of chemicals [22,23]. Thus, biofilms decrease bacterial susceptibility to antimicrobial agents, promoting bacterial tolerance and/or resistance and favoring the failure of eradication therapies [24].

*P. aeruginosa* is often co-isolated with other microbial species sharing the same environment. While its interactions—including both cooperation and competition—with classical pathogens *Burkholderia* spp. and *S. aureus* have been extensively studied [25,26,27,28,29,30,31,32], the available information regarding interactions with emerging pathogens such as *Achromobacter* spp. and *S. maltophilia* is still limited. Despite the reported co-isolation of *P. aeruginosa* and *Achromobacter* spp. from sputum samples and the increasing number of patients becoming chronically infected with the latter [6,9,33,34], thus far, only one recent study evaluated the occurrence of inter-species interactions between these two microorganisms, reporting that *P. aeruginosa* biofilm formation can be affected by *A. xylosoxidans* [35]. To evaluate the possible evolution of such interplay and its underlying mechanisms, in the present study, we assessed the ability of *Achromobacter* spp. isolates to affect the biofilm of *P. aeruginosa* strains sharing the same lung environment during long-term chronic co-infections and searched for genetic features of virulence possibly associated with the competition ability.

## 2. Results

*P. aeruginosa* and *Achromobacter* spp. clinical isolates were longitudinally collected from two CF patients chronically co-infected for over 9 years: patient A since 1996, and patient B since 1999. For each patient, the two species were isolated from the same sputum sample twice: in 2005 and 2008 from patient A, and in 2008 and 2014 from patient B. The general information on each isolate is presented in Table 1. The genotypic relatedness of longitudinal isolates was verified by core genome similarity: *P. aeruginosa* isolates from patients A and B showed 82% and 83% similarity, respectively, while *Achromobacter* spp. isolates from both patients showed 87% similarity. While all *Achromobacter* spp. isolates had been initially identified as *A. xylosoxidans* [36], a recent phylogenetic analysis reclassified the isolates from patient A as *A. insuavis* [37]. The first *A. insuavis* strain collected from this patient, named isolate A1, was previously classified as a hypermutator [36]. *P. aeruginosa* isolates from patient A belong to the DK08 clone type, sampled from multiple patients at the Copenhagen CF Center [38].

### 2.1. Phenotypic Variations

**Figure 1:**
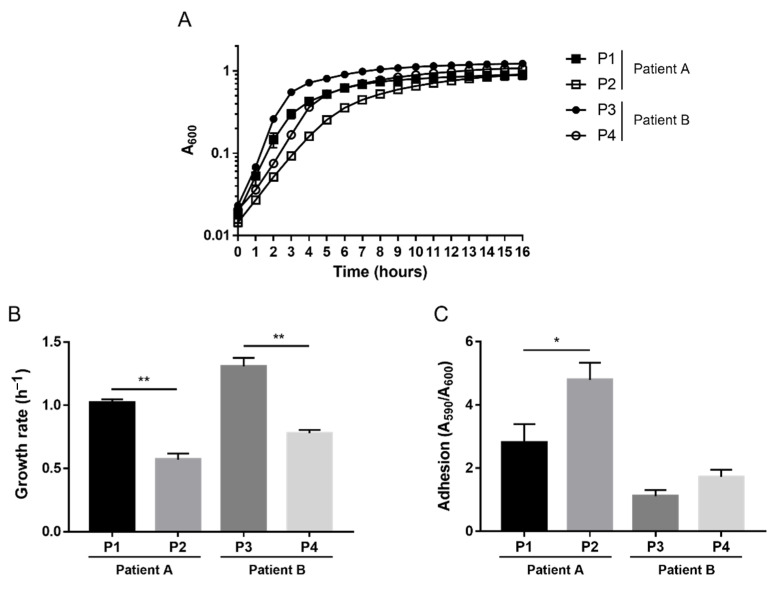
Growth curves (**A**), growth rate (**B**) and adhesion (**C**) of *P. aeruginosa* isolates. For growth curves, absorbance at 600 nm (A_600_) was measured every hour for the first 16 h (**A**). The growth rate was calculated from the exponential phase of growth curves (**B**). Adhesion was measured by absorbance of crystal violet-stained surface-attached bacteria (A_590_) divided by absorbance of planktonic bacteria (A_600_) (**C**). Each value represents the mean ± SEM of three experiments. Statistical analysis was performed by *t*-test, * *p* < 0.05, ** *p* < 0.01.

To investigate possible phenotypic variations within the same host over time, we evaluated features such as growth rate and adhesion, which are known to often undergo modifications during bacterial adaptation into the CF lung. We previously observed that no significant changes in terms of growth rate and adhesion ability occurred over time within the longitudinal *Achromobacter* spp. isolates from the two patients [36]; growth curves are shown in Figure S2. On the contrary, *P. aeruginosa* isolates underwent the phenotypic evolution known to occur during CF chronic infection: the growth rate significantly diminished over time in both patients, while the adhesion ability increased significantly in patient A (Figure 1).

### 2.2. Effects of Achromobacter spp. on P. aeruginosa Adhesion

**Figure 2:**
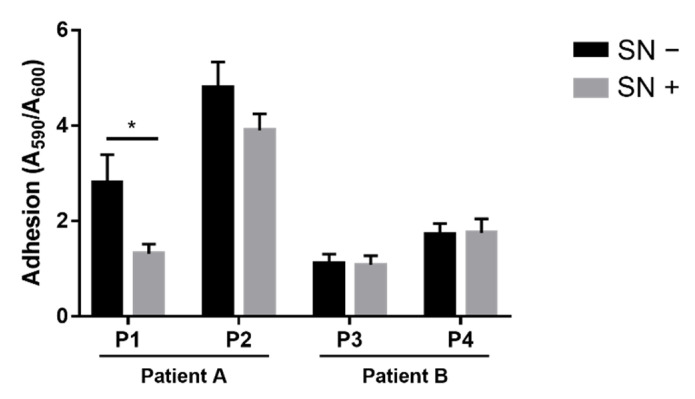
*P. aeruginosa* adhesion in the absence and presence of the co-isolated *Achromobacter* spp. culture supernatant (SN−, SN+). Adhesion was measured by crystal violet staining of surface-attached bacteria divided by A_600_ of planktonic bacteria. Each value represents the mean ± SEM of 3 experiments. Statistical analysis was performed by *t*-test, * *p* < 0.05.

To investigate whether *Achromobacter* spp. isolates exhibited competition against *P. aeruginosa*, we first evaluated the potential effects on its adhesion. This is an essential ability for biofilm formation, which is considered a key feature for the successful colonization of CF lungs by *P. aeruginosa* and other bacterial species. The adhesion of each *P. aeruginosa* strain was measured in the absence and presence of the culture supernatant collected from the co-isolated *Achromobacter* sp. strain. The culture supernatant contained all the exoproducts released during bacterial growth, including virulence factors; e.g., we previously observed a higher protease activity in an A1 culture supernatant than in supernatants from the other *Achromobacter* spp. isolates [36]. The P1 isolate showed a significantly lower adhesion ability when grown in the presence of A1 exoproducts (Figure 2). No such inhibitory effect was exhibited by the culture supernatants of the other *Achromobacter* spp. strains on their *P. aeruginosa* co-isolates, nor on the *P. aeruginosa* laboratory strain PAO1 (Figure S3).

### 2.3. Effects of Achromobacter sp. on P. aeruginosa Biofilm Formation

**Figure 3:**
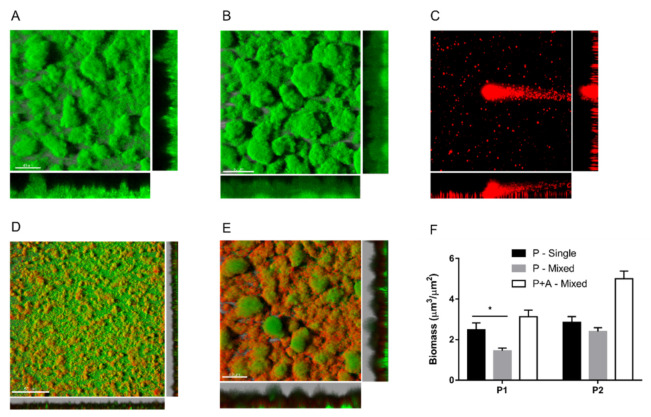
Single-species biofilms formed by *P. aeruginosa* P1 (**A**) and P2 (**B**) isolates, representative image of *Achromobacter* sp. biofilm structures (**C**), mixed biofilms formed by P1 + A1 (**D**) and P2 + A2 (**E**) strains and biomass quantification (**F**). Biofilms were grown in a flow chamber system for 5 days and monitored by confocal microscopy. *P. aeruginosa* isolates were tagged with GFP (green), and *Achromobacter* sp. cells were counterstained with Syto62 (red). *P. aeruginosa* biomass in single and mixed biofilms (P-Single, P-Mixed) and total biomass of mixed biofilms (P+A-Mixed) were calculated using Comstat2 software. Each value represents the mean ± SEM of 3 experiments. Statistical analysis was performed by the Mann–Whitney test, * *p* < 0.05.

To investigate whether the A1 isolate could also inhibit *P. aeruginosa* biofilm formation, mixed biofilm cultures were grown in a flow chamber system for up to 5 days. To distinguish the two species, *P. aeruginosa* strains were tagged with the green fluorescent protein (GFP), and the fluorescence emission was checked (Figure S1). In single-species cultures, as expected, *P. aeruginosa* isolates could form big, stable aggregates firmly attached on the glass surface (Figure 3A,B). On the contrary, *Achromobacter* sp. strains showed a poor adhesion ability on glass, forming sporadic, unstable aggregates characterized by the scattering and dispersal of planktonic cells (Figure 3C). When the two microbes were cultured together, *Achromobacter* sp. could adhere and form mixed biofilms with *P. aeruginosa*. However, the *Achromobacter* sp. A1 isolate interfered with the biofilm formation of the co-isolated *P. aeruginosa* P1 strain. As shown in Figure 3D, P1 aggregates are smaller in the presence of A1, as also confirmed by the *P. aeruginosa* biomass quantification (Figure 3F). Such inhibitory effect was not observed in mixed biofilms formed by their longitudinal co-isolates A2 and P2 (Figure 3E).

### 2.4. Genetic Variants in Achromobacter sp. Virulence Genes

Previously, we performed variant analysis of *Achromobacter* spp. isolates and observed that the A2, A3 and A4 genomes harbor no or few mutations, with a predicted high impact on protein function, while various frameshift mutations and a stop gain were detected in the A1 genome [36]. To find genetic evidence that could explain the observed inhibitory effect of the A1 isolate on the adhesion and biofilm formation of the P1 strain, we evaluated whether some of the genetic variants in the A1 genome involve genes related to virulence and inter-species competition. Interestingly, in A1, but not in the A2 genome, we detected the presence of a type VI secretion system tip protein, the VgrG gene, whose product is reported to bind antibacterial effectors targeting essential cell structures during inter-species competition between Gram-negative bacteria such as *Acinetobacter baumannii* [39]. Moreover, we found a stop gain in the HlyD family efflux transporter periplasmic adaptor subunit gene, whose product is a component of type I secretion systems involved in the secretion of virulence factors such as toxins and proteases [40]. When compared to the reference genome, this A1 gene results in a slightly shorter protein (−15 amino acids), while for the A2 strain, the predicted length of the same gene product is largely reduced (−129 amino acids). Although no data are available regarding the effect of these variants on the protein function, we can hypothesize that the HlyD protein is more likely functional in the A1 rather than the A2 isolate.

## 3. Discussion

For a long time, CF lung infection has been studied and treated as a disease caused by a single pathogen, while, nowadays, we are aware of its polymicrobial nature [41,42]. Within microbial communities, intra- and inter-species interactions can take place and potentially influence the course of the infection [18,19]. Interactions involving the emerging pathogens *Achromobacter* spp. have only rarely been studied, probably because the clinical relevance of this microorganism became evident more recently. Nonetheless, their increased prevalence in CF and their frequent co-isolation with other pathogens such as *P. aeruginosa* suggest that *Achromobacter* spp. likely have to compete for space and nutrients [43]. In the case of chronic co-infections, where cohabitation can last for a long time, the evolution of such interplays might as well be part of the bacterial adaptation processes known to occur in CF airways. In the present investigation, we focused on *Achromobacter* spp.’s behavior towards *P. aeruginosa* within biofilm communities and observed both competition and cohabitation interplays during chronic co-infections.

As regards the biofilm mode of growth, *Achromobacter* spp. are motile (swimming) via long, peritrichous flagella but lack twitching motility [44,45], which can contribute to the development of a surface-attached biofilm as it may help in stabilizing interactions with the surface [46]. Indeed, the poor adhesion ability of this microorganism in vitro (on polymeric surfaces within 48 h) has been reported [45]. A reduction in surface attachment over time during infection was also shown in sequential CF isolates, in association with the acquisition of mutations in genes with a presumptive role in surface adhesion [47]. However, some studies highlighted *Achromobacter* spp.’s ability to adhere on hydrogel contact lenses [48,49] and to form unattached or loosely attached aggregates held together by polysaccharides forming a peripheral shell around the bacterial cells [45,50]. Our current and previous [36] results confirm the poor adhesion ability of *Achromobacter* spp. on polystyrene and glass, and the formation of loosely attached aggregates characterized by the scattering and dispersal of planktonic cells. Interestingly, when cultured with *P. aeruginosa*, the two microbes could form mixed biofilms, suggesting that *Achromobacter* spp.’s adhesion might be enhanced on biotic surfaces.

Concerning inter-species interactions, we observed that only the A1 isolate has inhibitory effects against the co-isolated *P. aeruginosa* strain, interfering with its adhesion ability and affecting its biofilm formation capability. Conversely, the clonal strain A2 later isolated from the same patient, as well as two *Achromobacter* sp. strains longitudinally collected from another patient, did not show similar competitive behavior against their *P. aeruginosa* co-isolates. Thus far, only one recent study evaluated the occurrence of inter-species interactions between *Achromobacter* sp. and *P. aeruginosa* CF isolates, reporting that *P. aeruginosa* biofilm formation can be affected by *A. xylosoxidans* [35]. The isolate showing competition in our study belongs to a different species, *A. insuavis*, suggesting that this behavior might be common to various species of the genus. Interestingly, this isolate was previously classified as a hypermutator [36], and the isolates that Menetrey and colleagues observed to affect the *P. aeruginosa* biofilm were morphologically different clones collected from the same sputum sample of a chronically infected patient [35], a situation often exactly associated with the presence of hypermutators [51]. Although no genomic data are available from their study—limiting the evaluation of the hypermutation contribution—the association of this evolutionary mechanism with *Achromobacter* spp.’s competitive behavior should be further verified.

Investigating the genomic features that could be implicated in the observed competition, in the genome of the A1 isolate, we identified genetic variants supporting its higher virulence. Only in this isolate, we detected a type VI secretion system tip protein, the VgrG gene, whose product is known to be involved in inter-species competition between Gram-negative bacteria [39]. Additionally, *VgrG* paralogues have also been reported to regulate bacterial motility, biofilm formation and protease production in *Aeromonas* sp. [52]. Moreover, the A1 strain harbored a likely functional *HlyD* gene, while a deleterious variant was present in the genome of its clonal late isolate. HlyD is essential for the secretion of the RTX hemolytic toxin HlyA from *Escherichia coli* [53] and seems to be involved in the protease secretion mechanisms of P. aeruginosa [54]. We previously observed a higher protease secretion from the A1 strain in comparison to its clonal late isolate and to isolates from patient B [36], further supporting a higher expression of virulence traits in this isolate that could be involved in the observed competition against *P. aeruginosa*. Additionally, the genes related to the quorum sensing system were checked in all the *P. aeruginosa* isolates for the presence of variants, but no mutations were detected that could indicate down-regulation of this system.

Interestingly, 3 years after the A1 and P1 strains’ isolation, their competitive interplay evolved towards a more indolent cohabitation or even cooperation—similar to the situation observed in patient B—which might represent a survival advantage. Although major adaptations of bacteria causing CF chronic infections are likely to happen during the early stage of chronic infection, while in the late stage, the situation is supposedly more stable, we observed variations in the interplay between two microorganisms in the late stage of a chronic co-infection, suggesting that adaptive mechanisms are still ongoing. In the later stage, isolates A2 and P2 could also grow together in mixed biofilm communities, supporting the possibility that close microbial interactions might occur between them. Indeed, bacterial proximity within biofilm communities can favor social exchanges of signal molecules and genetic elements, influencing many aspects of the community itself such as the microbial composition, nutrient availability, and antibiotic resistance [21]. Although the occurrence of close microbial interactions within the CF airway has not been demonstrated, in this particular case, their relevance should be considered: *Achromobacter* spp. are usually rich in mobile genetic elements carrying antibiotic resistance [11,55], whose exchange with and acquisition by other microbes such as *P. aeruginosa* might influence the course of infection and the outcome of antibiotic therapies.

In conclusion, despite our observations being limited to restricted mechanisms on a small number of selected strains, our results show that both inter-species competition and cohabitation are represented during chronic co-infections in CF airways, and evolution of these interplays can happen at the late stages of chronic infection. Furthermore, we provided insights on virulence mechanisms that could be involved in *Achromobacter* spp.’s competitive abilities. Future studies on a larger scale, involving more strains from more patients, are needed to better understand the interplay between competition and adaptation in the lungs of CF patients. Further mechanisms involved in inter-species interactions should also be explored, such as regulation of quorum sensing and secretion of specific virulence factors or metabolic by-products. In addition, evaluating interactions involving other microbial species would increase insights into the extent and complexity of such interplays and their contribution to the clinical outcome. This highlights the importance and necessity of further studies with a larger number of isolates, encouraging further research on this subject.

## 4. Materials and Methods

### 4.1. Bacterial Isolates

Four clinical isolates of *Achromobacter* spp. and *P. aeruginosa* were collected from two CF patients followed in the CF clinic at Rigshospitalet in Copenhagen, Denmark. The use of the stored bacterial isolates was approved by the local ethics committee at the Capital Region of Denmark (Region Hovedstaden) with the registration number H-4-2015-FSP. *P. aeruginosa* and *Achromobacter* spp. were isolated from the same sputum sample twice from each patient: in 2005 and 2008 from patient A, and in 2008 and 2014 from patient B.

### 4.2. Growth Curves

Bacterial strains were plated on LB agar and incubated at 37 °C for 24–48 h. One colony was picked from the plate and inoculated in 10 mL LB medium, with shaking at 37 °C overnight. Optical density at 600 nm (OD_600_) was measured using a spectrophotometer, cultures were diluted to 0.05 OD/mL in LB medium and 150 µL/well was incubated in a 96-well plate for 20–24 h, with shaking at 37 °C. Using an automated plate reader, OD_600_ was measured every 20 min. Growth rate was calculated using GraphPad Prism 7.0.

### 4.3. Culture Supernatant Collection

*Achromobacter* spp. strains were plated on LB agar and incubated at 37 °C for 24–48 h. One colony was picked from the plate and inoculated in 10 mL LB medium, with shaking at 37 °C for 16 h. OD_600_ was measured, and cultures were diluted to 0.1 OD/mL in 10 mL of LB medium. After shaking at 37 °C for 16 h, cultures were diluted to 1 OD/mL and centrifuged at 7000× *g* for 30 min at 4 °C. Supernatants were collected and sterile filtered.

### 4.4. Adhesion Assay

Bacterial strains were plated on LB agar and incubated at 37 °C for 24–48 h. One colony was picked from the plate and inoculated in 10 mL LB medium, with shaking at 37 °C overnight. OD_600_ was measured using a spectrophotometer, cultures were diluted to 0.05 OD/mL in LB medium and 150 µL/well was incubated in a 96-well plate for 20–24 h at 37 °C. After measuring OD_600_, wells were washed twice with water to remove unattached cells, and surface-attached cells were stained with 0.1% crystal violet solution for 15 min. Wells were rinsed and washed three times with water and then dried for 1–2 h. Thirty percent acetic acid was added, incubated at room temperature for 15 min, and absorbance at 590 nm was measured. Adhesion measured by crystal violet staining (absorbance at 590 nm) was normalized on growth (absorbance at 600 nm). In competition assays, *P. aeruginosa* adhesion was measured in the presence/absence of 10% *Achromobacter* spp. culture supernatant.

### 4.5. GFP Tagging

*P. aeruginosa* strains were tagged with a mini-Tn7 construct carrying gentamycin resistance and GFPmut3b genes under the control of the growth-dependent *E. coli* ribosomal promoter *rrnB* P1 [56]. The construct was introduced in *P. aeruginosa* by conjugative transfer as described by Choi and Schweizer [57]. Briefly, recipient *P. aeruginosa* strains were mixed with *E. coli* donor and helper strains (pRK2013 and pNTS2), and a drop of bacterial suspension was placed in the center of an LB agar plate and incubated at 37 °C overnight. Transconjugants were selected by plating bacteria on LB agar containing gentamycin and trimethoprim. Mini-Tn7 insertion was checked by colony PCR using PTn7R and PglmSF primers [57]. To assess the growth-dependent fluorescence emission, *P. aeruginosa* GFP-tagged strains were cultured in a 96-well plate for 12 h, with shaking at 37 °C, while fluorescence (excitation 475 nm, emission 520 nm) and OD_600_ were measured every 20 min using an automated plate reader.

### 4.6. Biofilm Formation Assay

A flow chamber system was assembled and sterilized following the protocol from Tolker-Nielsen and Sternberg [58]. Briefly, 250 µL of bacterial cultures (0.05 OD/mL) was injected in each flow cell channel. Flow cells were left upside-down for an hour without flow to let bacteria attach on the cover glass and then were turned and incubated at 30 °C for up to 5 days with flow (A10 minimal medium added with MgCl_2_, CaCl_2_ and trace metals). Biofilm formation was observed by confocal laser scanning microscopy for GFP-tagged cells and Syto62 staining of total cells. For statistical analysis, at least 7 pictures/channel were taken, homogeneously distributed along the channel. Pictures were visualized and elaborated using Imaris 7.4 software. Biomass was calculated using Comstat2 software [59].

### 4.7. Genomic Analysis

Whole genome sequencing and assembly were performed as previously described [36]. Sequences have been deposited at EMBL under the projects n. PRJEB35058 (*Achromobacter* spp. sequences) and PRJEB40978 (*P. aeruginosa* sequences). Sequence data can be found with the experiment accession numbers ERX3614542 (strain A1), ERX3614543 (strain A2), ERX3614548 (strain A3, previously called B1), ERX3614549 (strain A4, previously called B2), ERS5248144 (strain P1), ERS5248145 (strain P2), ERS5248146 (strain P3) and ERS5248147 (strain P4). Genotypic relatedness among longitudinal isolates was verified by checking core genome similarities obtained using the Harvest-OSX64-v1.1.2 suite [60]. Variant analysis was performed as previously described [36]. Briefly, two types of variant analysis were carried out: the first by aligning sequence reads to the reference genome, and the second by aligning them to the de novo assembly of the longitudinal isolate from the same patient. In the first case, the annotated genomes *A. xylosoxidans* NH44784-1996 and *P.* *aeruginosa* PAO1 (RefSeq accessions: GCF_000967095.2 and GCF_000006765.1) were used as reference genomes. Bowtie 2 v2.3.4.1 [61] was used for performing reads alignment, and the SnpEff v4.3t toolbox [62] was used to annotate variants and predict their functional effects. Only variants supported by a minimum of 20 reads were retained.

## Figures and Tables

**Table 1:** Identification and time of isolation of each clinical strain.

Patient	Isolate	Species	Year of Isolation	Other Characteristics
A	A1	*A. insuavis*	2005	Hypermutator
A	P1	*P. aeruginosa*	2005	DK08 clone type
A	A2	*A. insuavis*	2008	
A	P2	*P. aeruginosa*	2008	DK08 clone type
B	A3 *	*A. xylosoxidans*	2008	
B	P3	*P. aeruginosa*	2008	
B	A4 *	*A. xylosoxidans*	2014	
B	P4	*P. aeruginosa*	2014	

* A3 and A4 isolates were called B1 and B2 in a previous study [36].

## Data Availability

Sequencing data are publicly available at EMBL under the projects n. PRJEB35058 (*Achromobacter* spp. sequences) and PRJEB40978 (*P. aeruginosa* sequences).

## Supplementary Materials

The following are available online at https://www.mdpi.com/article/10.3390/pathogens10080978/s1, Figure S1: Growth and fluorescence emission curves of GFP-tagged *P. aeruginosa* isolates; Figure S2: Growth curves of *Achromobacter* spp. isolates, Figure S3: Adhesion of *P. aeruginosa* PAO1 strain in absence and presence of the culture supernatants collected from the clinical *Achromobacter* spp. isolates.
