# Supplementary material for: Adaptive Interactions of Achromobacter spp. with Pseudomonas aeruginosa in Cystic Fibrosis Chronic Lung Co-Infection
Source: Pathogens. 2021 Aug 3;10(8):978. doi: 10.3390/pathogens10080978 (PMC8400197; doi:10.3390/pathogens10080978)
Supplement: Supplementary file 1 [file pathogens-10-00978-s001.zip › pathogens-1265493-SI.pdf]

Supplementary materials:

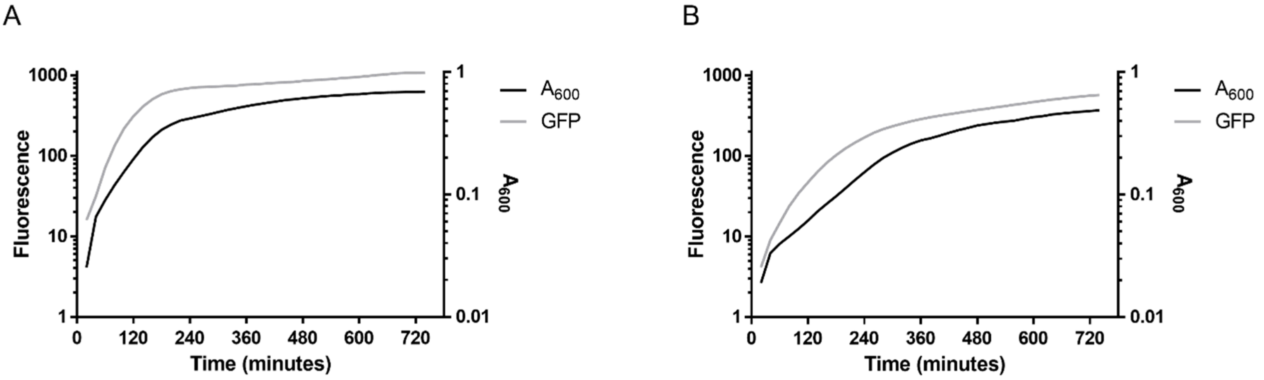

Figure S1. Growth and fluorescence emission curves of GFP-tagged P1 (**A**) and P2 (**B**) isolates. GFP fluorescence (excitation 475 nm, emission 520 nm) and A_{600} were measured every 20 min for 12 h.

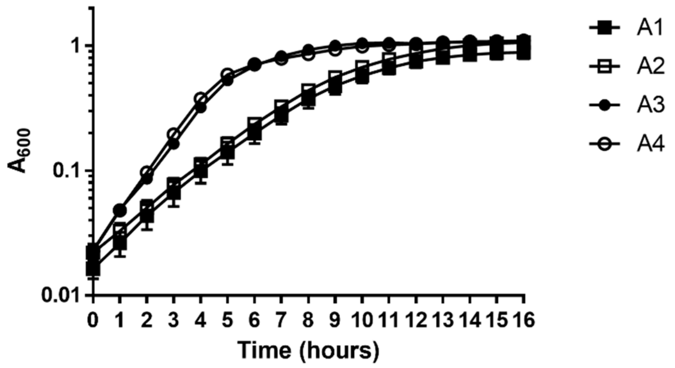

Figure S2. Growth curves of *Achromobacter* spp. isolates. A_{600} was measured every hour for 16 h.

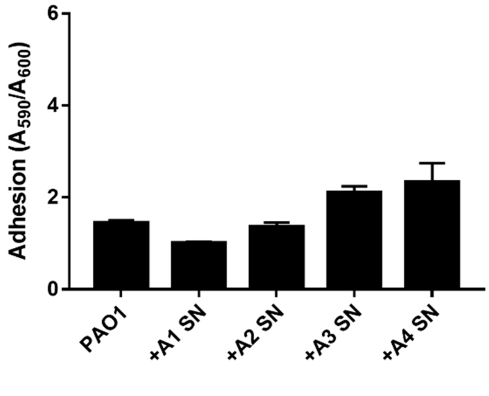

Figure S3. Adhesion of *P. aeruginosa* PAO1 strain in absence and presence of the culture supernatants (SN) collected from the clinical *Achromobacter* spp. isolates (A1, A2, A3 and A4). Adhesion was measured by crystal violet staining of surface-attached bacteria (A_{590}) divided by absorbance of planktonic bacteria (A_{600}).
